# Subjective Perceptions and Conceptualisations of Ageing and Disability in Ghana: An Exploratory Sequential Mixed‐Methods Study

**DOI:** 10.1002/puh2.70324

**Published:** 2026-08-10

**Authors:** Doris Akosua Tay, Mumuni Abu, Fidelia Dake, Delali Margaret Badasu

**Affiliations:** ^1^ University of Ghana Accra Ghana

**Keywords:** ageing stereotypes, ageism, conceptualisation, disability perceptions, Ghana, subjective perception, sub‐Saharan Africa

## Abstract

**Background:**

In Ghana and sub‐Saharan Africa, there is limited understanding of how people's subjective perceptions influence the broader conceptualisation of ageing and disability. Cultural and social factors play a critical role in shaping these perceptions, but existing research has not adequately explored this relationship. This study addresses this gap by using the newly developed Subjective Perception of Ageing and Disability Scale (SPADS) and the Conceptualisation of Ageing and Disability Scale (CADS) to examine how individual beliefs and functional abilities interact with cultural and social contexts to shape views on ageing and disability in Ghana.

**Methods:**

An exploratory sequential mixed‐methods research design explored how ageing symptoms are associated with disability among residents of Dodowa in the Shai Osudoku District, Greater Accra region. The initial qualitative phase involved 14 participants, followed by a quantitative phase with 175 participants. Themes from the qualitative study informed the development of instruments for the quantitative study. SPADS and CADS developed through Cronbach's *α* analysis and exploratory factor analysis. An Independent samples *t*‐test, analysis of variance (ANOVA) and structural equation modelling assessed subjective perceptions of ageing and disability and their broader conceptualisation, respectively.

**Findings:**

The findings indicate a close relationship between subjective perceptions of ageing and disability in Ghana. Ageing symptoms identified in the qualitative phase—cognition, physical attributes, sensory impairment, and potential disability—were associated with disability. Most participants associated disability with ageing symptoms as reflected in their SPADS responses. SPADS was a strong predictor of CADS, with a positive relationship between the two scales. Most participants conceptualised ageing as being inherently linked to disability.

**Conclusion:**

Ageing is predominantly perceived as being associated with disability. The findings suggest a lack of adequate knowledge about ageing and disability. Educational campaigns are recommended to improve understanding of these phenomena. Future research should explore longitudinal changes in perception and rural–urban differences.

## Graphical abstract

This mixed‐methods study demonstrates that subjective perceptions of ageing strongly influence broader conceptualisations of ageing and disability in Ghana. The findings highlight the importance of culturally informed approaches to promoting healthy ageing and reducing misconceptions about disability in later life.

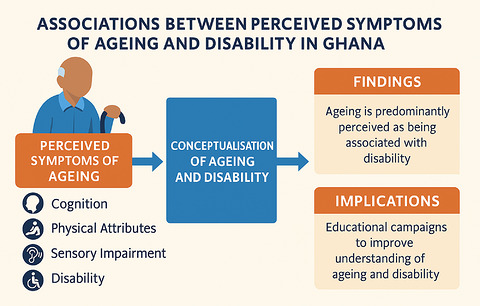

## 1 Introduction

Definitions of old age vary, influenced by factors such as chronological age, biological changes and social roles [1, 2]. African societies often emphasise social roles over biological changes [3, 4]. In Ghana, for example, age is perceived as a continuous process dependent on life conditions and social status [4, 5]. Language further shapes perceptions of ageing in Ghana, with terms used to describe older adults carrying connotations of weakness and incapacity [6].

The interpretation of disability is context‐dependent, influenced by age, gender, education, and personal experiences [7, 8]. Regarding age, older adults may either or not identify as disabled due to normalised functional difficulties associated with ageing [7, 8, 9]. Younger adults tend to hold negative stereotypes of ageing, associating it with sickness, whereas older adults often view ageing positively [7, 10, 11, 12, 13]. Disability's definition varies across models [14], notably the medical and social models. The medical model views disability as an individual's functional limitation resulting from impairment [15], whereas the social model attributes it to societal barriers [16, 17, 18, 19]. The biopsychosocial perspective integrates both models, considering biological, individual and social factors. The International Classification of Functioning, Disability and Health (ICF) emphasises the interaction between an individual's health condition and contextual factors, broadening the understanding of disability beyond individual limitations [20, 21].

The perception of ageing and disability is deeply influenced by societal norms and individual experiences [22, 23, 24, 25, 26]. In many cultures, ageing is associated with notions of successful ageing, which encompass physical, mental and social well‐being [27, 28, 29]. However, these ideals often exclude those with disabilities, leading to a narrow view of what constitutes successful ageing. Older adults may internalise these societal perceptions, equating age‐related health issues with disability, even when they may not meet clinical definitions of disability.

The relationship between subjective perceptions of ageing and broader conceptualisations of ageing and disability can be understood through several complementary theoretical perspectives. Building on the conceptual definitions outlined above, this study draws on the Theory of Planned Behaviour, Self‐Perception Theory, Social Identity Theory, Lifespan Development Theory and Symbolic Interactionism to explain how individual beliefs, social interactions and cultural contexts shape perceptions of ageing and disability. Together, these frameworks provide the theoretical basis for examining how subjective perceptions of ageing may influence broader conceptualisations of ageing and disability. These theoretical perspectives are particularly relevant in Ghana, where cultural norms, family structures and social beliefs strongly influence experiences and understandings of ageing and disability.

Ghana's population, which is approximately 30.8 million, is predominantly young, with those aged 18 and older comprising 58.2% of the population [30]. Despite this youthful demographic, the proportion of older adults (aged 65 years and above) is on the rise, currently representing 4.3% of the population [30, 31]. The prevalence of disability is significantly higher among older adults, with 22.2% of individuals aged 65 and older experiencing some form of disability, compared to less than 10% in younger age groups [32]. Presently, 8% of the Ghanaian population faces difficulties in performing daily activities [30].

This demographic evolution underscores the necessity of understanding how ageing and disability are perceived within Ghana's culturally rich environment, where societal norms and beliefs heavily influence these perceptions. Most older adults in Ghana are integrated into the family unit, providing care to the younger generation and receiving care themselves [31]. These interactions shape subjective perceptions of ageing and disability among the younger generation.

Despite extensive global research on perceptions of ageing and disability, few studies have investigated these issues in sub‐Saharan Africa, a region where culture, language and social norms strongly shape ageing experiences. This study provides needed evidence from Ghana, where no prior research has empirically tested the relationship between subjective perceptions and broader conceptualisations using locally validated tools.

By introducing and applying context‐specific scales, this research offers a systematic analytical framework through which ageing and disability can be examined in a region where such tools have been lacking. By positioning Ghana within the broader ageing literature, the study contributes to ongoing global conversations by demonstrating both parallels and culturally specific nuances in how ageing and disability are understood.

### 1.1 Study Objective

This study, therefore, examines the relationship between subjective perceptions of ageing and disability and how they are conceptualised more broadly in the Ghanaian context. Using the newly developed Subjective Perception of Ageing and Disability Scale (SPADS), and the Conceptualisation of Ageing and Disability Scale (CADS), this research provides a unique approach to understanding how cultural and social influences affect views on ageing and disability. This study is necessary to contribute knowledge to bridge the gap in ageing studies in sub‐Saharan Africa.

### 1.2 Hypotheses

The study hypothesises that

1. Higher SPADS scores, indicating stronger subjective perceptions that ageing is associated with disability, will be positively associated with higher CADS scores, reflecting a stronger conceptualisation of ageing as inherently linked to disability.
2. Lower SPADS scores, indicating weaker subjective perceptions that ageing is associated with disability, will be associated with lower CADS scores, reflecting a conceptualisation of ageing and disability as distinct phenomena.

## 2 Methods

### 2.1 Study Design

The study employed an exploratory sequential mixed research method. This approach involved a case study qualitative inquiry of factors that shaped subjective perception of ageing and its association with disability. This was followed by cross‐sectional quantitative analysis of the relationships between the SPADS, together with socio‐demographic variables and the CADS. The integration of both qualitative and quantitative components involved using qualitative findings to inform the development of quantitative research instruments.

### 2.2 Participants and Sampling

The study comprises participants, including young, middle‐aged and older adults, categorised, respectively, as 20–39, 40–59 and 60 and over. Conducted in Dodowa, the capital town of the Shai Osudoku District in the Greater Accra Region of Ghana, the research employed purposeful sampling for the qualitative phase. Saturation guided the determination of sample size, with no new issues emerging after the 14th respondent. For the quantitative study, a spatially systematic sampling approach was employed to select participants, as a complete household listing was unavailable. The process began by randomly selecting the first house at the junction of Matekye Street, which connects to the main Dodowa road and the Shai Osudoku District office. From this point, every second house was selected along the street to ensure structured and unbiased household selection.

This approach helped mitigate challenges, such as empty houses or ineligible residents, thereby improving access to the target population. To enhance representation, all eligible individuals within selected households were included in the study. Although this was not a purely probability‐based sampling method, the systematic household selection strategy improved geographical coverage and facilitated recruitment in the absence of a complete household sampling frame. Consequently, the findings should be interpreted with appropriate caution, as some selection bias may have occurred, and the results may not be fully representative of the wider Ghanaian population.

The participants were interviewed in their homes. Interviews lasted for approximately 40 min, although some interviews went beyond an hour because the participants engaged interviewers in conversations on the topic. The respondents who were eligible for the study in every second house were interviewed by the researcher and research assistants in Dangme, Twi and English. A total of 175 participants were systematically sampled from a population of individuals aged 20 years and above (6633) residing in Dodowa.

### 2.3 Data Collection and Tools

The qualitative study employed face‐to‐face and telephone interviews. Researcher journals and field notes supplemented data collection, with two Dangme‐fluent research assistants aiding in interviews. Semi‐structured interview guides, aligned with study objectives, guided discussions. Interviews occurred between 26 August and 16 September 2021, lasting 35 min to an hour.

Quantitative data were collected via questionnaire using Kobo Toolbox. The questionnaire encompassed socio‐demographic variables and qualitative themes, mirroring ageing concepts, WHODAS 2.0 and ICF concepts [25, 33]. Four trained research assistants administered the survey from 21 September to 26 2022, lasting about 40 min.

### 2.4 Pretesting of Research Instruments

The instruments were pretested before use for both qualitative and quantitative studies on 10th August 2021 and 15 to 17 September 2022, respectively. Eleven participants from Shiashie in East Legon and Sebrepor in the Kpone Katamanso district partook in the pretesting of the research instruments.

### 2.5 Scoring, Response Options and Interpretation

The study included both ordinal and binary response formats, requiring a systematic approach to scoring. Ordinal variables were measured using a three‐point Likert scale, where participants responded to statements about ageing and disability: 3 = Agree (strong association with ageing and disability), 2 = Neutral (some association) and 1 = Disagree (no association). Binary variables required participants to select factors they associated with ageing and disability. These responses were scored as 3 = Selected (participant believes this factor is associated with ageing with disability) and 1 = Not selected (participant does not associate this factor with ageing with disability). This scoring structure ensured a consistent quantification of perceptions, capturing both graded and categorical associations.

In this study, higher scores on the SPADS and CADS scales, along with a positive direction of the relationship between them, indicate that participants conceptualise ageing as being inherently linked to disability. Conversely, lower scores and a negative direction of the relationship suggest that participants view ageing as distinct from disability. See Tables S3 and S4 for SPADS and CADS items with scoring response options.

### 2.6 Measures

#### 2.6.1 Dependent Variable: CADS

The dependent variable was theorised using the CADS developed from three scales dubbed the Subjective Perception Of Ageing And Disability Scale (SPADS), the medical factors scale (MFS) and the contextual factors scale (CFS) from interviews with participants from Dodowa, Ghana. The SPADS, MFS and CFS were rigorously developed through a process informed by qualitative themes and quantitative analysis using Cronbach's *α* analysis and exploratory factor analysis (EFA).

Key domains, such as cognition, physical attributes, sensory impairment and the perception of disability during ageing informed the development of SPADS items. Themes for medical factors included diseases, health decline and performance of ADLs and IADLs (life activities, self‐care activities and mobility). Themes, such as fear, social participation, beliefs and experience, made up contextual factors. Participants described issues in relation to these themes as either ageing with or without disability. These qualitative themes informed the development of SPADS, MFS and CFS items. The instrument development process is illustrated in Figure S1, and a detailed mapping of qualitative themes to CADS items is provided in Supporting Information File 1.

An initial reliability analysis was conducted on 41 items (15 from SPADS, 16 from MFS and 10 from CFS), yielding Cronbach's *α* of 0.963, indicating acceptable internal consistency. However, one item (curses) from SPADS had a negative inter‐item correlation with ‘experience from other family members’ (−0.037) (suggesting it did not measure the same underlying construct as that item). ‘Curses’ were removed. Cronbach's *α* remained at 0.963. See Table S1 for the 41 variables analysed using Cronbach's *α* and EFA.

An EFA was conducted to examine the latent structure of the CADS and to evaluate the dimensionality of the construction. This was done using the programme FACTOR version 12.02.01, selecting polychoric correlation analysis due to the presence of both binary and ordinal variables [34, 35]. This method was chosen because Pearson correlation can bias factor loadings when variables are not continuous. A pre‐factor analysis of all 40 variables resulted in the removal of 15 items from the pool. The remaining 25 items underwent EFA.

An EFA was performed with 25 variables; however, the polychoric correlation matrix was found to be non‐positive definite, indicating serious multicollinearity and data irregularities among the items. Specifically, the smoothing algorithm applied to correct this problem (Sweet Smoothing) destroyed 58.9% of the covariance structure, an unacceptable level of distortion that renders the factor solution unreliable.

To achieve a stable and interpretable factor structure, nine problematic variables were, therefore, removed on the basis of the following criteria:

1. Very low variance, indicating limited discrimination power.
2. Highly skewed or kurtotic response distributions, violating the assumptions for polychoric correlation estimation.
3. Variables involved in smoothing adjustments that destroyed a large proportion of covariance information.

After eliminating these variables, the remaining 16 items yielded a positive definite correlation matrix suitable for EFA, resulting in a more robust and theoretically meaningful factor solution.

An EFA using robust diagonally weighted least squares (RDWLS) extraction and Promax rotation revealed a unidimensional structure, with all 16 items loading significantly on a single factor. The scree plot and parallel analysis confirmed the retention of one factor, which accounted for 56.1% of the total variance (eigenvalue = 8.98). The Kaiser–Meyer–Olkin (KMO) measure of sampling adequacy was 0.906, indicating excellent suitability for factor analysis. Bartlett's Test of Sphericity was significant (*χ* ^2^ = 1932.3, *p* < 0.001), confirming that the correlation matrix was not an identity matrix and therefore appropriate for factor analysis. The scale's internal consistency was excellent, with EAP reliability = 0.963 and a factor determinacy coefficient = 0.981, indicating highly reliable factor scores.

Factor loadings ranged from 0.490 (shouts) to 0.920 (pain), with communalities spanning 0.240–0.847. The strongest contributors to the latent construct were items reflecting physical and functional limitations (e.g., pain, sits to bath, fear of inability to perform life activities and fear of mobility challenges), whereas behavioural and emotional indicators also showed meaningful loadings. The item *knowledge from personal growth* loaded positively (0.667), suggesting that even positive ageing experiences may be interpreted within the broader context of disability.

Bootstrapped bias‐corrected and accelerated (BCa) confidence intervals confirmed the stability of the factor loadings, with all intervals excluding zero. Model fit indices further supported the robustness of the factor solution: RMSEA = 0.000, CFI = 0.999 and NNFI = 1.033, all indicating excellent fit. The EAP reliability of the factor scores was 0.963, and the factor determinacy index was 0.981, suggesting high precision in estimating the latent trait. See Table 1 for the results of the factors with their loadings and Table 4 for the distribution of participants’ scores on the CADS scale.

**TABLE 1:** Conceptualisation of Ageing and Disability Scale (CADS) loading matrix.

Variable	Factor loading	Communality	BCa confidence interval
Strange behaviour	0.699	0.489	(0.358, 0.883)
Shouts	0.490	0.240	(0.232, 0.728)
Quarrelsome	0.552	0.305	(0.268, 0.782)
Thinking slows	0.856	0.733	(0.435, 0.939)
Memory loss	0.709	0.502	(0.369, 0.873)
Pain	0.920	0.847	(0.464, 0.992)
Sits to bath	0.847	0.717	(0.446, 0.999)
Assisted to washroom	0.606	0.367	(0.304, 0.843)
Cannot cook	0.630	0.397	(0.316, 0.851)
Rests between walks	0.773	0.598	(0.400, 0.922)
Knowledge from personal growth	0.667	0.445	(0.319, 0.925)
Fear of inability to perform life activities	0.909	0.825	(0.452, 1.084)
Fear of mobility challenges	0.841	0.707	(0.430, 1.003)
Fear of disease	0.512	0.262	(0.239, 0.732)
Fear of disability	0.646	0.417	(0.348, 0.901)
Fear of witchcraft accusation	0.599	0.358	(0.298, 0.956)

Abbreviation: BCa, bias‐corrected and accelerated.

Although the conceptualisation of ageing and disability incorporates multiple cognitive, physical, behavioural, and contextual dimensions, the EFA indicated that these domains collectively represented a single latent construct. This finding reflects the empirical structure observed within the study population and should not be interpreted as suggesting that ageing and disability are inherently unidimensional concepts.

#### 2.6.2 Independent Variable: Subjective Perception of Ageing and Disability Scale (SPADS)

The SPADS items were developed through a rigorous qualitative process. It captures perceptions of ageing symptoms and examines how these are variably associated with disability, highlighting distinct yet interconnected perceptions. Participants’ indications of whether they associate the symptoms with disability allowed for a differentiated analysis of ageing and disability perceptions. Themes, identified from the qualitative data, spanning cognition, physical attributes, sensory impairment and possibility of disability, were utilised to construct the questionnaire for the quantitative study. A detailed mapping of qualitative themes to SPADS items is provided in Supporting Information File 1.

**FIGURE 1:**
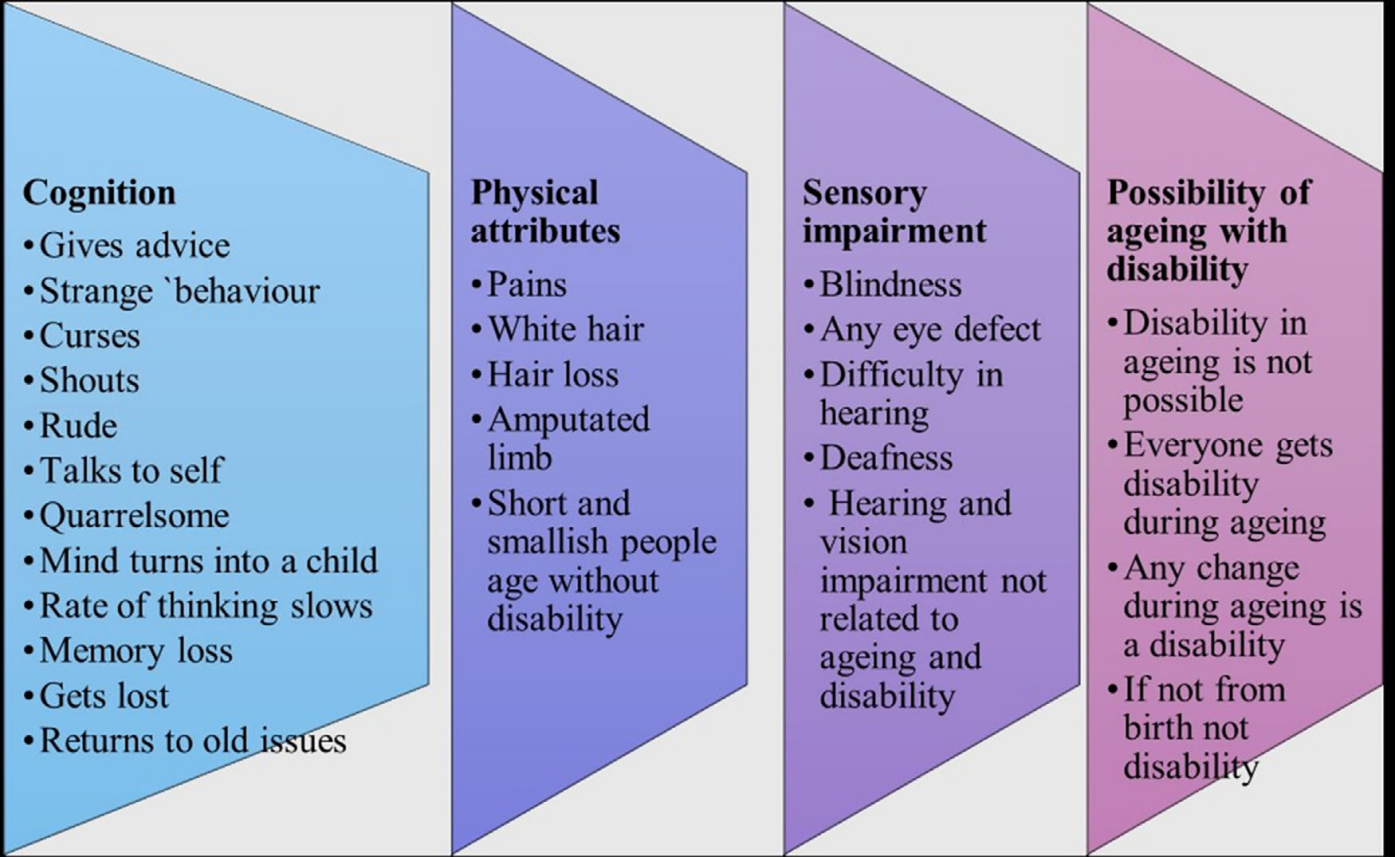
First 26 items for reliability analysis.

Through Cronbach's *α* analysis and EFA, the SPADS was developed and designated as the independent variable. A total of 26 items derived from the qualitative findings were incorporated into the quantitative questionnaire. The reliability analysis of these items yielded Cronbach's *α* of 0.803, ensuring internal consistency. The 26 items and their corresponding themes are depicted in Figure 1.

The inter‐item correlation matrix revealed negative correlations among multiple items. Initially, four (4) items were eliminated to address this issue. For example, the item *blindness* exhibited negative inter‐item correlations with several variables, including *strange behaviour* (−0.241), *shouts* (−0.161), *curses* (−0.013) and *rude* (−0.069). As a result, blindness was excluded from the item pool. A reliability test conducted on the remaining 22 items showed Cronbach's *α* of 0.856. Subsequently, seven (7) additional items were removed due to their negative correlation with other variables. Ultimately, 11 items that did not adequately measure the ‘subjective perception of ageing and disability’ were deleted. See Table S2 for the 26 initial items and the retained 15 items.

An EFA was conducted to examine the underlying structure of the 15 items comprising the SPADS. Prior to extraction, the suitability of the data for factor analysis was established. The KMO measure of sampling adequacy was 0.823, indicating good sampling adequacy, and Bartlett's test of sphericity was significant (*χ* ^2^ = 1532.45, *p* < 0.001), confirming that the correlation matrix was factorable.

Both the scree plot and parallel analysis supported a single‐factor solution, with the first factor yielding an eigenvalue of 9.246 and accounting for 66.0% of the total variance. Subsequent eigenvalues were below 1.0, and none exceeded the 95th percentile of simulated random data, further supporting the unidimensionality of the scale.

Factor loading ranged from 0.55 (Hearing difficulty) to 0.94 (Memory loss), with the majority exceeding 0.70, indicating strong relationships with the underlying latent construct. Items, such as *mind turns into a child*, *thinking slows*, *memory loss*, *gets lost*, *pains*, *white hair* and *return to old issues*, demonstrated particularly high communalities (>0.60), suggesting they were well explained by the factor. The scale's internal consistency was excellent, with EAP reliability = 0.977 and a factor determinacy coefficient = 0.989, indicating highly reliable factor scores.

All factor loadings were statistically significant (*p* < 0.001) with narrow 95% bootstrap confidence intervals, indicating stable estimates across resamples.

Model fit indices indicated that the one‐factor model fit the data extremely well (*χ* ^2^ = 42.76, *p* > 0.05 (0.645); RMSEA = 0.000; CFI = 0.999; GFI = 0.965; WRMR = 0.046). Collectively, these results provide evidence that the SPADS is a unidimensional, psychometrically sound measure capturing subjective perceptions of ageing and disability, as reflected in behavioural, cognitive and physical manifestations associated with ageing. Table 2 displays the SPADS variables along with their factor loadings and communalities from the EFA.

**TABLE 2:** SPADS loading matrix.

Variable	Factor loading	Communality	BCa confidence interval
Strange behaviour	0.740	0.548	(0.516, 0.873)
Curses	0.407	0.165	(0.235, 0.594)
Shouts	0.703	0.495	(0.351, 0.816)
Rude	0.582	0.339	(0.315, 0.728)
Talks to self	0.904	0.818	(0.520, 0.974)
Quarrelsome	0.708	0.502	(0.346, 0.799)
Mind turns into a child	0.915	0.838	(0.572, 0.978)
Thinking slows	0.927	0.858	(0.656, 0.982)
Memory loss	0.787	0.620	(0.592, 0.895)
Gets lost	0.671	0.450	(0.366, 0.807)
Pains	0.887	0.787	(0.605, 0.958)
White hair	0.867	0.751	(0.617, 0.938)
Hair loss	0.785	0.617	(0.583, 0.918)
Return to old issues	0.945	0.893	(0.758, 1.011)
Hearing difficulty	0.533	0.284	(0.313, 0.723)

Abbreviation: BCa, bias‐corrected and accelerated.

Although ageing and disability are conceptually multidimensional, the retained items consistently reflected a single underlying construct representing participants’ overall subjective perception of the relationship between ageing and disability. The emergence of a unidimensional structure suggests that, within this study population, these perceptions were experienced as a coherent construct rather than as distinct but unrelated domains.

#### 2.6.3 Socio‐Demographic Variables

The socio‐demographic characteristics considered in this study include age, sex, ethnic group, religious affiliation, marital status, education level, economic activity, number of children ever born alive, perceived wealth status, experience with older adults and the respondent's disability status.

Age was categorised into three groups: young adults (20–39 years), middle‐aged adults (40–59 years) and older adults (60 years and above). Sex was recorded as either male or female. Ethnic group was classified as Ga‐Dangme or other ethnic groups. Religious affiliation was categorised as Christianity, Islam or no religious affiliation. Marital status was grouped into currently married/in union, previously married or never married.

The highest level of education attained was categorised as never attended school, junior high school (JHS) or below and senior high school (SHS) or higher. Economic activity was classified as not engaged in any economic activity, engaged in economic activity with pay or engaged in economic activity without pay. Perceived wealth status was categorised as poor, middle or rich.

Experience with older adults was assessed on the basis of whether the respondent had ever lived with an older adult and whether that older adult had a disability. Finally, the respondent's own disability status was recorded as either yes or no.

### 2.7 Data Analysis

#### 2.7.1 Qualitative Data Analysis

ATLAS.ti 9 facilitated data organisation and analysis through the creation of a case study database comprising interview recordings, transcripts, field notes, reports and reflective memos. Interviews, predominantly in Dangme with some in Twi and English, were audio‐recorded and transcribed verbatim into English. A native Dangme speaker was consulted to verify the accuracy of translated meanings and preservation of participants’ intended expressions. The single Twi interview was translated by the bilingual interviewer, who checked the translation for conceptual equivalence.

Data analysis followed a constant comparative method [36], beginning with open coding using both inductive and in vivo techniques. Codes were refined through iterative comparison, merging similar codes and splitting complex ones into subcodes. Axial coding was used to group codes into categories, followed by theme integration to develop broader conceptual structures. Coding reliability was enhanced through memo writing, researcher journaling and repeated validation of codes and categories against participant quotations. Although formal intercoder reliability statistics were not computed, collaborative review and validation of codes and themes ensured consistency and conceptual alignment.

#### 2.7.2 Quantitative Data Analysis

SPSS version 22 was utilised for data analysis, FACTOR 12.02.01 facilitated the EFA, whereas JASP 0.95.3 was used for the structural equation modelling (SEM). Descriptive statistics were first used to characterise the study sample and summarise SPADS and CADS scores. Independent‐samples t‐tests and one‐way analysis of variance (ANOVA) were subsequently conducted to examine whether subjective perceptions of ageing and disability (SPADS) differed according to participants’ socio‐demographic characteristics. Finally, structural equation modelling (SEM) was used to test the study's primary hypotheses and conceptual framework by examining the relationship between subjective perceptions of ageing and disability (SPADS) and their broader conceptualisation (CADS). The analytical strategy therefore was guided by the study's conceptual model, which proposes that subjective perceptions of ageing and disability influence broader conceptualisations of these phenomena. SPADS was, therefore, specified as the primary predictor of CADS. In addition, the MFS and CFS were included to examine whether medical and contextual influences contributed to participants’ conceptualisations of ageing and disability beyond subjective perceptions. SEM was selected because it enabled the simultaneous estimation of these hypothesised relationships within a single analytical framework.

Significance was determined at *p* ˂ 0.05. Prior to estimating the SEM, a preliminary linear regression of CADS on SPADS, MFS and CFS was performed to evaluate model assumptions. Collinearity diagnostics indicated no multicollinearity (Tolerance = 0.397–0.544; VIF = 1.84–2.52; Condition Index < 13). Residuals were approximately normally distributed (*M* = 0.00, SD = 0.99) with no signs of heteroscedasticity. One case showed a standardised residual of −3.89, but its influence was negligible (all Cook's *D* < 1). These diagnostics confirmed model adequacy for SEM estimation. Data screening in SPSS indicated no missing values for the study variables; hence, the full sample (*N* = 175) was used in all analyses.

### 2.8 Theoretical Underpinning for Employing SPADS and CADS

Recent scholarship has highlighted persistent conceptual gaps in how ageing and disability are understood within both gerontological and disability studies. Reference [37], for instance, demonstrate that ageing research often frames disability mainly as functional decline linked to biological ageing, whereas disability studies emphasise socio‐material barriers, exclusion, and rights‐based perspectives. These parallel traditions have resulted in blurred boundaries in which ageing and disability are sometimes conflated or insufficiently differentiated. Such conceptual ambiguity limits empirical clarity and complicates policy responses, especially in regions like sub‐Saharan Africa where theoretical development remains emergent.

This study responds to these gaps by treating ageing and disability as distinct yet intersecting constructs. The SPADS and CADS scales were, therefore, developed to measure (a) subjective perceptions of ageing and disability and (b) broader conceptualisations of their relationship. This aligns with the growing call for analytical clarity in understanding how individuals interpret ageing‐related changes, how these interpretations link to disability, and how social, cultural and psychological factors influence these perceptions across diverse settings.

‘Successful ageing’ in this study refers to ageing characterised by positive psychosocial wellbeing, functional capacity and adaptive engagement with later life, rather than simply the absence of disease. This definition emphasises that individuals can experience ageing positively even in the presence of health conditions, and it provides a foundation for examining how subjective perceptions influence broader conceptualisations of ageing and disability.

Building on this conceptual grounding, the study draws on several theoretical perspectives that explain how subjective perceptions of ageing and disability (SPADS) may shape broader conceptualisations (CADS). These theories clarify the mechanisms through which attitudes, experiences and sociocultural contexts influence how ageing and disability are understood.

#### 2.8.1 Individual Perceptions (SPADS) → Broader Conceptualisations (CADS)

##### 2.8.1.1 Theory of Planned Behaviour [38]

This theory proposes that attitudes, subjective norms and perceived behavioural control influence intentions and behaviour. In this context, individuals’ attitudes towards ageing (measured by SPADS) are expected to influence how they conceptualise ageing and disability (measured by CADS). If people perceive ageing negatively, they might conceptualise ageing and disability in a more negative light. If SPADS scores show negative perceptions of ageing (with positive SPADS scores), it is expected that these individuals would have more positive CADS scores, reflecting a conceptualisation that is negative and associated with disability.

##### 2.8.1.2 Self‐Perception Theory [39]

Individuals infer their attitudes and beliefs by observing their own behaviour. Here, positive self‐perceptions (e.g., maintaining activity despite ageing reflected in negative SPADS) are expected to correspond with more positive conceptualisations of ageing and disability (negative CADS).

##### 2.8.1.3 Lifespan Development Theory [40]

This theory emphasises that development occurs across the lifespan and is shaped by biological, psychological and social factors. How individuals perceive ageing affects their adaptation strategies, which, in turn, influence broader conceptualisations. Positive perceptions of ageing (as measured by SPADS) should lead to adaptive strategies that are reflected in more positive conceptualisations of ageing and disability (as measured by CADS).

#### 2.8.2 Social Context and Interaction

##### 2.8.2.1 Social Identity Theory [41]

Individuals’ sense of self is shaped by their group memberships, including age cohorts. Positive identification with one's age group can foster positive perceptions of ageing and corresponding conceptualisations. Positive identification with the ageing process (as measured by SPADS) should lead to more positive conceptualisations of ageing and disability (as measured by CADS).

##### 2.8.2.2 Symbolic Interactionism [42]

This theory posits that people develop meanings of objects, events and concepts through social interaction. Ageing and disability are understood through the meanings individuals and society ascribe to them. These socially derived meanings influence broader conceptualisations (CADS), shaped by individual perceptions (SPADS). Interactions that reinforce positive perceptions of ageing (negative SPADS scores) should correlate with positive conceptualisations of ageing and disability (negative CADS scores) whereby disability is not associated with ageing.

##### 2.8.2.3 Social Constructionism [43]

This perspective asserts that social realities, such as ageing and disability, are constructed through cultural and societal norms rather than being purely biological or objective. Individual perceptions (SPADS) both influence and are influenced by these societal constructions (CADS).

Overall, this framework integrates individual, social and developmental perspectives to examine subjective experiences of ageing and disability in Ghana. It maintains the conceptual distinction between ageing and disability while providing a basis to explore how personal perceptions influence broader societal conceptualisations. This approach supports the study's aim of generating novel empirical insights in a region where ageing research is still emerging.

Moreover, these theoretical perspectives informed the specification of SPADS as the predictor construct and CADS as the outcome construct in the subsequent statistical analyses.

## 3 Socio‐Demographic Characteristics

### 3.1 Qualitative Study Participants

Table 3 displays the socio‐demographic characteristics of the participants for the qualitative study. Most of the participants were females with more than half of them in the middle‐age group. Only a few of the participants had never attended school with half of them either in union or currently married. Most of the participants previously lived with an older adult, although most of them lived with an older adult without a disability. For disability status of the respondents, the majority had no disability.

**TABLE 3:** Socio‐demographic characteristics of both qualitative and quantitative study participants.

Variable	Qualitative (*N* = 14) %	Quantitative (*N* = 175) %
Age		
20–39	28.6	37.7
40–59	50	32.6
6 days over	21.4	29.7
Sex		
Male	28.6	43.4
Female	71.4	56.6
Ethnic group		
Ga‐Dangme	93	73.7
All other ethnic groups	7	26.3
Religious affiliation		
Christianity	100	93.7
Islam	—	4.0
No religion	—	2.3
Marital status		
In union/currently married	50	43.4
Previously married	28.6	32.0
Never married	21.4	24.6
Highest educational level		
Never attended school	7	11.4
JHS/Middle or lower	64	53.7
SHS or higher	29	34.9
Economic activity		
No economic activity	35.7	4.0
Economic activity with pay	64.3	69.7
Economic activity with no pay	—	26.3
Children ever born alive		
No children	21.4	13.1
1 or more children	78.6	86.9
Perceived wealth status		
Poor	21.4	4.0
Middle	78.6	59.4
Rich	—	36.6
Ever live with older adult		
Yes	85.7	85.1
No	14.3	14.9
Older adult lived with had disability		
Yes	28.6	20
No	57.1	64
Respondent has disability		
Yes	85.7	84.6
No	14.3	15.4

Abbreviations: JHS, junior high school; SHS, senior high school.

### 3.2 Quantitative Study Participants

Over half of the respondents in the quantitative study were female, and various age groups were represented, with less than half falling into the middle‐aged, young and older adult categories. The majority had received some form of education, although a small percentage had never attended school. Marital status varied, with a significant portion currently married/in union, never married or previously married. Christianity was the predominant religious affiliation, followed by Islam and a small percentage with no religious affiliation.

Regarding children ever born alive, a minority of respondents had no children, whereas the majority identified as Ga‐Dangme ethnically. Economic activity engagement was common, with most respondents engaged in paid economic activities, although a notable percentage was not engaged in any economic activity.

Perceptions of wealth status varied, with a majority perceiving themselves as middle class and a significant minority considering themselves poor. Many respondents had experience living with an older adult, although fewer had lived with an older adult with a disability. A large majority of respondents reported no disability, whereas a significant minority indicated having a disability. Overall, these characteristics provide a snapshot of the diverse socio‐demographic landscape of the Shai Osudoku District. See Table 3 for the results.

Overall, the sample captured participants from diverse demographic and socioeconomic backgrounds, providing a broad range of perspectives on ageing and disability. The predominance of Ga‐Dangme participants reflects the ethnic composition of the study area, whereas the relatively high proportion of participants engaged in paid economic activity and those with previous experience living with older adults provides an appropriate context for examining perceptions of ageing and disability. Nevertheless, these characteristics should be considered when interpreting the broader generalisability of the findings beyond the study community.

### 3.3 Constructs That Constitute Subjective Perception of Ageing Among the Participants in the Qualitative Study

The qualitative study delved into the components comprising the subjective perception of ageing and disability among the participants. Key themes that emerged from the data include cognition, physical attributes, sensory impairment and the possibility for disability during ageing. These findings indicate that individuals’ subjective perceptions of ageing and its correlation with disability were influenced by factors such as cognitive abilities, specific physical traits, the presence of sensory impairments, and whether ageing was perceived to coincide with disability or not.

#### 3.3.1 Theme: Cognition

The data revealed two main categories related to cognition: communication and cognitive attributes. Communication had both verbal and non‐verbal behaviours, whereas cognitive attributes pertained to mental faculties. Negative communication was associated with both disability and non‐disability contexts, whereas positive communication was linked to ageing without disability. These patterns were consistent across different age groups, although some variations were noted among middle‐aged adults. For instance, although some middle‐aged adults considered talking excessively as a sign of disability in ageing, others did not share this view. Additionally, cognitive attributes were perceived as part of ageing with or without disability. Some attributes, such as a regression of the mind to a childlike state, were interpreted differently by participants. Cognitive decline was another pattern identified, with differing perspectives on its universality and causes. Although some attributed cognitive changes to ageing with disability, others attributed them to external factors such as poverty or divine intervention. These divergent views were observed within age groups rather than across different age cohorts. Some middle‐aged and older adults viewed cognitive decline as a disability associated with ageing, whereas others did not share this perspective.

#### 3.3.2 Theme: Physical Attributes

The study revealed distinct patterns in how participants perceived physical changes associated with ageing, shaping their understanding of ageing with and without disability. For some, disability in ageing was equated with the onset of bodily discomfort, including pain in the knees and overall weakness, coupled with the visual markers of ageing such as hair turning white. This perception linked the experience of physical ailments to the concept of ageing with disability. In contrast, others related disability in ageing to the persistence of health issues over time, irrespective of attempts to manage them. One perspective emphasised the inability to recover from health problems as individuals aged, leading to the perception of ageing with disability.

Conversely, some participants who described ageing without disability highlighted the importance of body size and structure in shaping their perception. They noted that individuals with smaller physiques seemed to maintain their ability to perform daily tasks as they aged, contrasting this with the potential for physical limitations in those with larger body sizes. These insights underscore the complexity of how individuals perceive ageing and disability, revealing the multifaceted nature of these constructs and their intersection with physical attributes.

#### 3.3.3 Theme: Sensory Impairment

Sensory impairment, particularly vision and hearing loss, emerged as a significant theme in the study, with participants expressing varying views on whether it constitutes ageing with or without disability. Most of the participants, across all age groups, characterised vision and hearing impairment as ageing with disability. This consensus was consistent across age groups, indicating little variation in perceptions based on age. However, within age groups, there were some nuanced differences in how individuals conceptualised sensory impairment. For instance, one participant described ageing with an eye problem as a disability, citing an older adult who was able to read without spectacles even at the age of 80. This individual contrasted their own experience of eye strain while reading with the older adult's ability to read effortlessly, emphasising the possibility of ageing without disability in relation to sensory impairment.

In contrast, another participant categorised having an eye problem as a disease rather than a disability, highlighting a distinction between impairment and disability. This perspective challenges the notion that sensory impairment necessarily equates to disability and suggests a more nuanced understanding of age‐related health issues. The prevailing view among participants, however, was that vision and hearing issues are indicative of ageing with disability. This consensus aligns with the medical model of disability, which conceptualises impairment as a precursor to disability. Overall, whereas there were some divergent perspectives on the relationship between sensory impairment and disability, the dominant narrative among participants emphasised the challenges and limitations associated with ageing‐related sensory decline, underscoring the importance of addressing these issues in the context of disability.

#### 3.3.4 Theme: Possibility of Ageing With Disability

Participants in the study expressed diverse views regarding the relationship between ageing and disability. Although some believe that disability is an inevitable part of ageing and is experienced by everyone, others disagreed, suggesting that disability may occur at any age and is not necessarily linked to ageing. These differing perspectives were evident across all age groups, with some participants asserting that disability must be present early in life before one can age with a disability, whereas others acknowledged the possibility of disability occurring during both ageing and earlier years. For example, one participant viewed disability as an intrinsic aspect of ageing, attributing it to factors beyond individual control such as fate or divine intervention. In contrast, another participant argued that disability is separate from the ageing process and may occur independently of it, emphasising the importance of contextual factors in shaping disability experiences.

These contrasting viewpoints reflect differing conceptualisations of ageing and disability, with some participants aligning with the medical model, which emphasises the biological aspects of disability, whereas others espouse a more social model perspective, which considers disability as a product of social and environmental factors. Some participants distinguished between the ageing process itself and the occurrence of disability, suggesting that disabilities acquired later in life should not be automatically attributed to ageing but may result from other influences. This nuanced understanding underscores the complex interplay between ageing, disability and broader sociocultural factors, highlighting the need for a holistic approach to addressing perceptions of disability in the context of ageing.

Taken together, these findings demonstrate that participants’ perceptions of cognitive decline, physical changes, sensory impairment and the possibility of ageing with disability reflect the complex ways in which subjective perceptions of ageing intersect with broader conceptualisations of disability. Rather than representing isolated themes, these findings collectively informed the development of the SPADS and CADS instruments and provided the conceptual foundation for the subsequent quantitative analyses, which examined whether these qualitatively identified perceptions were reflected in broader conceptualisations of ageing and disability.

Building on these qualitative findings, the quantitative phase examined whether the themes identified from participants’ narratives were reflected in the SPADS and CADS measures and whether subjective perceptions of ageing were associated with broader conceptualisations of ageing and disability.

### 3.4 Results for the Quantitative Study

#### 3.4.1 Distribution of Scores on the CADS

Table 4 presents the distribution of scores on the CADS. The mean score of 21.54 (SD = 6.98) falls slightly below the midpoint of the observed range (11.26–33.77), indicating that, on average, participants expressed moderate to moderately low associations between ageing and disability. The relatively large interquartile range (IQR = 12.27) further suggests considerable variation in how participants conceptualise the relationship between ageing and disability.

**TABLE 4:** Distribution of Conceptualisation of Ageing and Disability Scale (CADS) and SPADS scores.

Number of respondents	175	
	CADS	SPADS
Mean	21.5375	23.2182
Standard deviation	6.98450	7.46089
Median	20.6160	23.0840
Minimum	11.26	11.36
Maximum	33.77	34.08
Percentiles		
25	15.7790	15.3920
50	23.0840	20.6160
75	30.3160	27.6570

The 25th percentile score (15.39) indicates that approximately one quarter of participants viewed ageing and disability as largely distinct concepts, showing weak association between the two. The median score (20.62) reflects a moderate perception, suggesting that half of the participants perceived some connection between ageing and disability. The 75th percentile score (27.66) indicates that the upper quartile of participants held stronger beliefs that ageing is closely associated with disability.

Overall, the distribution shows that although perceptions vary widely, a substantial proportion of participants associate ageing with some level of disability, reflecting diverse conceptualisations within the population.

#### 3.4.2 Distribution of Scores on the Subjective Perception of Ageing and Disability Scale

Table 4 presents the distribution of scores on the Subjective Perception of Ageing and Disability Scale (SPADS). The mean score of 23.22 (SD = 7.46) lies near the midpoint of the observed range (11.36–34.08), indicating a moderate subjective perception of ageing and disability among participants. The relatively wide IQR (=14.54) reflects substantial variation in individual perceptions.

The 25th percentile score (15.78) shows that approximately one quarter of participants reported low subjective perceptions, suggesting they view ageing as less strongly linked with disability. The median score (23.08) suggests that half of the participants held moderate perceptions, seeing some connection between ageing and disability. The 75th percentile score (30.32) indicates that a quarter of participants expressed stronger subjective associations between ageing and disability, reflecting a tendency to view disability as an expected or integral part of ageing.

#### 3.4.3 Conceptualisation of Ageing and Disability by Socio‐Demographic Characteristics: Results of the Independent Samples *T*‐Test and ANOVA

Independent samples *t*‐tests were conducted to examine differences in SPADS scores across selected demographic and experiential variables. The analysis revealed no significant difference in SPADS scores between males (*M* = 23.84, SD = 7.44) and females (*M* = 22.74, SD = 7.48), *t*(173) = 0.96, *p* > 0.05, *d* = 0.15. Similarly, there was no significant difference in SPADS scores between Ga‐Dangme (*M* = 23.17, SD = 7.22) and other ethnic groups (*M* = 23.35, SD = 8.18), *t*(71.49) = −0.13, *p* > 0.05, 95% CI [−2.90, 2.54], *d* = −0.02. This indicates a negligible difference in perceptions of ageing and disability across ethnic groups and sex. The results also indicated no significant difference between participants who had children and those who did not (*t*(173) = −0.52, *p* > 0.05, 95% CI [−3.90, 2.28], *d* = −0.11). This suggests that participants’ perceptions of ageing and disability did not differ on the basis of whether they had children. There was no significant difference between those who had ever lived with an older adult and those who had not (*t*(173) = −1.16, *p* > 0.05, 95% CI [−4.97, 1.29], *d* = −0.25). Participants who had lived with older adults (*M* = 22.94, SD = 7.56) reported slightly lower perceptions of ageing‐associated disability than those who had not (*M* = 24.79, SD = 6.77), but the effect was small and non‐significant. Participants who had lived with an older adult with a disability (*M* = 25.76, SD = 6.41) scored significantly higher on the SPADS than those who had lived with an older adult without disability (*M* = 22.13, SD = 7.72), *t*(67.53) = 2.77, *p* ˂ 0.05, 95% CI [1.02, 6.23], *d* = 0.49. This indicates that exposure to disability in older adults was associated with stronger perceptions of ageing as being linked with disability.

Results showed that participants with a disability (*M* = 25.89, SD = 7.17) had significantly higher SPADS scores than those without disability (*M* = 22.73, SD = 7.43), *t*(173) = 2.04, *p* ˂ 0.05, 95% CI [0.10, 6.21], *d* = 0.43. This suggests that respondents living with disability are more likely to associate ageing with disability compared to those without disability. Table 5 shows the results.

**TABLE 5:** Independent samples *t*‐test results in SPADS.

Socio‐demographic variable		*N*	Mean	Std. deviation	*p* value
Sex	Male	76	23.8383	7.44000	0.337
	Female	99	22.7421	7.47957	
Ethnic group	Ga‐Dangme	129	23.1717	7.21865	0.897
	All other ethnic groups	46	23.3485	8.18492	
Children ever born alive	None	27	22.5309	7.58189	0.604
	One or more	148	23.3436	7.45781	
Ever lived with an older adult	Yes	149	22.9447	7.56301	0.247
	No	26	24.7855	6.76816	
Older adult had disability	Yes	35	25.7587	6.41359	0.007
	No	112	22.1346	7.72067	
Respondent has disability	Yes	27	25.8891	7.16786	0.043
	No	148	22.7309	7.43319	

A one‐way ANOVA was conducted to examine differences in SPADS scores across age groups (20–39 years, 40–59 years and 60 years and above). The results indicated no statistically significant differences among the groups, *F*(2, 172) = 2.64, *p* > 0.05, *η* ^2^ = 0.03. Although not significant, the pattern of means suggested that participants aged 60 years and above (*M* = 21.61) tended to score slightly lower on the SPADS than those aged 20–39 years (*M* = 24.12) and 40–59 years (*M* = 24.67), suggesting a weaker association between ageing and disability among the oldest participants. Post hoc comparisons using the Games–Howell test showed no significant pairwise differences between age categories (all *p* > 0.05).

The results showed no statistically significant differences by participants’ educational attainment, *F*(2, 172) = 0.63, *p* > 0.05, *η* ^2^ = 0.01. Post hoc comparisons indicated no significant pairwise differences between any of the educational categories (all *p* > 0.05). The small and non‐significant effect size suggests that participants’ perceptions of ageing and disability were generally similar regardless of educational level.

The analysis by economic activity revealed no statistically significant differences across the three groups, *F*(2, 172) = 1.39, *p* > 0.05, *η* ^2^ = 0.02. Post hoc comparisons indicated that mean SPADS scores did not significantly differ between participants with no economic activity, those engaged in paid economic activity, and those engaged in unpaid economic activity, all *p* > 0.05. These results suggest that participants’ perceptions of ageing and disability were relatively similar regardless of their economic activity status.

A statistically significant difference among the groups was observed for marital status, *F*(2, 172) = 4.33, *p* ˂ 0.05, *η* ^2^ = 0.05, indicating a small‐to‐moderate effect of marital status on perceptions of ageing and disability. Post hoc comparisons revealed that participants who were separated, divorced or widowed had significantly higher SPADS scores than those who had never been married, *p* ˂ 0.05. This suggests that individuals who had experienced marital dissolution or widowhood were more likely to associate ageing with disability than those who had never married. No significant differences were observed between those in union/currently married and the other groups (*p* > 0.05).

Also, the analysis revealed no statistically significant differences for perceived wealth status, *F*(2, 172) = 1.14, *p* > 0.05 *p* = 0.323, *η* ^2^ = 0.01. Post hoc comparisons confirmed that mean SPADS scores did not significantly differ between participants who perceived themselves as poor, middle class, or rich (all *p* > 0.05).

There were no statistically significant differences based on religious affiliation, *F*(2, 172) = 1.25, *p* = 0.288, *η* ^2^ = 0.01. Post hoc comparisons indicated that mean SPADS scores were statistically similar among Christians, Muslims and participants with no religious affiliation (all *p* > 0.05). Table 6 presents the results.

**TABLE 6:** Analysis of variance (ANOVA) results in SPADS.

Socio‐demographic variables		Sum of squares	df	Mean square	*F*	*p* value
Age	Between groups	288.531	2	144.266	2.641	0.074
	Within groups	9397.152	172	54.635		
	Total	9685.683	174			
Highest level of education	Between groups	70.251	2	35.125	0.628	0.535
	Within groups	9615.433	172	55.904		
	Total	9685.683	174			
Economic activity engagement	Between groups	154.149	2	77.075	1.391	0.252
	Within groups	9531.534	172	55.416		
	Total	9685.683	174			
Marital status	Between groups	464.440	2	232.220	4.332	0.015
	Within groups	9221.243	172	53.612		
	Total	9685.683	174			
Perceived wealth status	Between groups	126.615	2	63.308	1.139	0.323
	Within groups	9559.068	172	55.576		
	Total	9685.683	174			
Religious affiliation	Between groups	139.143	2	69.571	1.253	0.288
	Within groups	9546.541	172	55.503		
	Total	9685.683	174			

#### 3.4.4 Results of the Structural Equation Modelling

The structural equation model (SEM) in Table 7 examined the relationship between SPADS and CADS, while controlling for variables such as the two other latent predictors (MFS and CFS) and socio‐demographic variables that might confound this relationship. The study hypothesised

1. Higher SPADS scores, indicating stronger subjective perceptions that ageing is associated with disability, will be positively associated with higher CADS scores, reflecting a stronger conceptualisation of ageing as inherently linked to disability.
2. Lower SPADS scores, indicating weaker subjective perceptions that ageing is associated with disability, will be associated with lower CADS scores, reflecting a conceptualisation of ageing and disability as distinct phenomena.

**TABLE 7:** SEM results.

Predictor	Estimate (*β*)	SE	*z* value	*p* value	95% CI
SPADS	0.412	0.024	17.27	<0.001	[0.365, 0.459]
MFS	0.272	0.021	13.02	<0.001	[0.231, 0.313]
CFS	0.436	0.020	22.08	<0.001	[0.398, 0.475]
Age 40–59	0.090	0.040	2.24	0.025	[0.011, 0.169]
Age 20–39	0.082	0.046	1.79	0.073	[−0.008, 0.172]
Male	0.074	0.031	2.39	0.017	[0.013, 0.135]
Other covariates	—	—	—	ns	—

*Note:* Residual variance for CADS = 0.034 (SE = 0.004, *p* < 0.001). Predictor variances were standardised (0.994). Residual covariances: SPADS‐MFS = 0.695, SPADS‐CFS = 0.661, MFS‐CFS = 0.543.

Abbreviations: CFS, contextual factors scale; MFS, medical factors scale.

The structural equation model was specified as just‐identified (df = 0), resulting in perfect fit indices by design. Interpretation therefore focuses on the magnitude and significance of structural paths. Fit indices were AIC = −60.67, BIC = −10.03, *χ* ^2^ = 0.000 and *N* = 175, indicating no model misfit. The SEM results showed that SPADS and the two other latent predictors significantly and positively influenced CADS scores. The SPADS (*β* = 0.412, SE = 0.024, *p* < 0.001) was a strong predictor, confirming that individuals with more negative subjective perceptions of ageing and disability were more likely to conceptualise ageing as accompanied by disability. Similarly, MFS (*β* = 0.272, SE = 0.021, *p* < 0.001) significantly predicted CADS, suggesting that medical limitations and experiences of pain or dependency play a key role in shaping this conceptualisation. The CFS (*β* = 0.436, SE = 0.020, *p* < 0.001) was the strongest predictor of CADS.

Among demographic variables, middle‐aged adults (40–59 years; *β* = 0.090, SE = 0.040, *p* < 0.05) and male participants (*β* = 0.074, SE = 0.031, *p* < 0.05) had significantly higher CADS scores, suggesting that these groups were more likely to view ageing as inherently linked to disability. Younger adults (20–39 years) showed a marginal effect (*β* = 0.082, *p* > 0.05, whereas education, ethnicity, marital status and religious affiliation were not significant predictors of CADS.

The residual variance for CADS was small but significant (0.034, SE = 0.004, *p* < 0.001), indicating that most of the variance in CADS scores was accounted for by SPADS and the two other predictor scales. All predictor variances were standardised (0.994). Moderate‐to‐strong residual covariances were observed among them—SPADS–MFS = 0.695, SPADS–CFS = 0.661 and MFS–CFS = 0.543 demonstrating shared variance among subjective, medical and contextual domains.

Overall, the SEM results confirm that subjective perceptions, medical experiences and contextual beliefs are all integral to how ageing and disability are conceptualised in this context. Although the SPADS remains an important predictor, its influence is best understood in relation to the physical limitations and sociocultural fears captured by the MFS and CFS. The consistent positive direction of effects across all three predictors supports the theoretical proposition that, within this population, ageing is widely conceptualised as being accompanied by disability.

Taken together, the quantitative findings support and extend the qualitative evidence by demonstrating that the themes identified during the qualitative phase are reflected in participants’ broader conceptualisations of ageing and disability.

**FIGURE 2:**
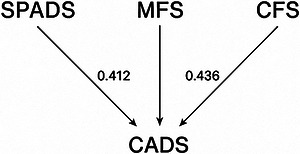
SEM of CADS. CFS, contextual factors scale; MFS, medical factors scale.

Figure 2 illustrates the predictive relationships among the SPADS, the MFS and the CFS as determinants of CADS. Standardised path coefficients are displayed on each arrow. All paths were statistically significant (*p* < 0.001). Double‐headed arrows represent residual covariances among the three predictor constructs. The model was saturated (df = 0) and demonstrated a perfect fit by design (AIC = −60.67, BIC = −10.03, *χ* ^2^ = 0.000).

## 4 Discussion

The qualitative findings of this study align with previous research, revealing patterns in subjective perception of ageing and how it influences conceptualisation of ageing and disability. Cognitive function played a significant role in participants’ subjective perception of ageing as in other countries [44, 45, 46, 47, 48]. Good cognitive function was seen as indicative of ageing without disability, whereas cognitive decline and bodily weakness were associated with the ageing process, as shown in a study from Ghana [49].

Physical attributes were essential in the participants’ subjective perception of ageing. Pain and physical limitations for instance were commonly associated with ageing with disability, whereas grey hair was generally perceived as a natural part of ageing. These are similar to findings in studies by [6, 13, 50, 51, 52]. Pain was particularly associated with disability due to its link with mobility impairment, as reported by older adults in Ghana [53].

Sensory impairment, particularly vision impairment and hearing difficulties, were linked to disability. This reflects varied perspectives on ageing and disability across different contexts. The recent Population and Housing Census in Ghana revealed that vision impairment had the highest prevalence among six domains of difficulty in performing activities [30]. This perception likely stems from the common association of vision issues with disability among the participants. Blindness, vision impairment and hearing difficulty are associated with ageing and disability in other societies [6, 45, 54, 55].

The participants’ views ranged from perceiving ageing as inherently associated with disability to rejecting such associations. This highlights the complex interplay between individual experiences and societal perceptions. The biopsychosocial model of disability provided a framework for understanding these diverse perspectives. Most of the participants aligned with the medical model, whereas others rejected it in favour of the social model. Additionally, participants distinguished between the ageing process and the process of ageing, emphasising that disability in old age should not necessarily be attributed to the ageing process itself, but rather to external factors. This reflects how some Ghanaians associate cognitive, physical and sensory impairments with disability in old age.

The quantitative study results reveal that the SPADS is a strong predictor of CADS, indicating that most of the participants inclined to negative perceptions of ageing and disability conceptualised ageing as being with disability. This suggests a strong association between people's individual perceptions of ageing and disability and broader conceptualisations. The univariate analysis also showed a dominant subjective perception that associated symptoms of ageing with disability.

The findings are consistent with the Theory of Planned Behaviour, which proposes that attitudes influence broader beliefs and behavioural orientations, and with Self‐Perception Theory, which suggests that individuals derive broader understandings from their own experiences. Together, these theories provide the primary explanation for the observed relationship between subjective perceptions and conceptualisations.

The influence of contextual factors further suggests that these perceptions are not solely individual but are socially constructed through cultural norms, family interactions and shared beliefs. Symbolic Interactionism and Social Constructionism, therefore, complement the primary interpretation by explaining how meanings attached to ageing and disability are negotiated within Ghanaian society.

Social Identity Theory and Lifespan Development Theory provide additional support by highlighting how age‐group identification and adaptation across the life course may shape perceptions of ageing and disability. These theories enrich the interpretation but are secondary to the central relationship demonstrated between subjective perceptions and broader conceptualisations. The findings are consistent with the theoretical framework underpinning SPADS and CADS.

The demographic analysis revealed that variables, such as marital status, disability status and experience with older adults with disabilities significantly influenced SPADS scores. However, education, ethnicity, religious affiliation and economic activity did not show significant effects. This may reflect cultural homogeneity within the study population or measurement limitations in capturing nuanced differences. It also suggests that subjective perceptions are shaped more by relational and experiential factors than by structural ones, reinforcing the relevance of symbolic interactionism and contextual influences.

Age plays a significant role in shaping subjective perceptions of ageing and disability, aligning with previous research [45, 51] suggesting that some older adults perceive ageing as inherently linked to disability. However, as expected [9, 56], the study observed significant differences in ageing perceptions between males and females. Although previous studies [57] have highlighted ethnic differences in ageing perceptions, this study did not observe significant variations in subjective perceptions across different ethnic groups. Educational level also did not emerge as a significant predictor of ageing perceptions, unlike findings from previous research [57] indicating that lower educational levels are associated with more negative perceptions of ageing. Additionally, despite the common association between economic activity (employment) and perceptions of ageing [57, 58], this study did not find significant relationships between economic activity engagement and ageing perceptions.

Recent research in sub‐Saharan Africa highlights that older adults’ perceptions of disability are shaped by both physiological changes and sociocultural interpretations [59, 60, 61, 62, 63, 64, 65, 66]. For example, an interpretive phenomenological study in Nigeria [61] found that older adults often attributed disability to age‐related decline, illness or spiritual factors such as divine intervention or witchcraft, with corresponding coping strategies that reflected local beliefs and practices. Similarly, stroke survivors in urban Ghana [65] reported that daily functioning, social participation and mental health were deeply intertwined with lived experiences of disability in older/older‑aged populations in Ghana and helped contextualise how functional limitations influence subjective and societal conceptualisations.

National‐level evidence from the 2021 Ghana Population and Housing Census [62] confirms that sensory and physical impairments are the most prevalent forms of difficulty among older adults, particularly vision problems, which aligns with the dominant narratives in this study linking sensory impairment to disability. Collectively, these findings suggest that although certain aspects of ageing and disability may be universal, such as the association of cognitive and physical decline with functional limitations, the interpretation of these changes is highly context‐specific, shaped by cultural, spiritual and social frameworks. The present study extends this evidence by providing a systematic, psychometrically grounded quantification of subjective perceptions (via SPADS) and their influence on broader conceptualisations of ageing and disability (via CADS), demonstrating both alignment with and divergence from global and regional patterns.

Both hypotheses proposed in the study were supported by the findings. The magnitude of the association between SPADS and CADS suggests that subjective perceptions are not peripheral beliefs but are central to how ageing and disability are conceptualised within this population. This has practical implications because interventions aimed at promoting more positive perceptions of ageing may also influence broader societal understandings of disability, thereby reducing age‐related stereotypes and supporting healthier ageing.

Overall, the study confirms that ageing is widely conceptualised as being with disability in this Ghanaian context. The integration of theoretical frameworks with empirical findings underscores the multifaceted nature of ageing perceptions and highlights the importance of addressing both individual attitudes and societal narratives in interventions. Future research should explore longitudinal changes in perception and examine rural–urban differences to further contextualise these findings.

Although global research has long established associations between ageing symptoms and disability, these insights have rarely been explored in sub‐Saharan Africa, where cultural constructions of ageing differ significantly from those in high‐income regions. Recent studies from East Asia, Europe and the United States highlight similar patterns of cognitive and physical decline shaping perceptions of disability. However, these studies are conducted in sociocultural contexts with distinct family structures, health systems and social norms. By contrast, Ghanaian communities emphasise extended family roles, local spiritual beliefs and culturally specific interpretations of impairment (e.g., witchcraft accusations and moral expectations of elders). This study therefore fills an important void by demonstrating how global ageing themes manifest within a Ghanaian context, while also revealing unique sociocultural nuances.

These contextual differences likely explain why, although the broad association between ageing and disability is consistent with international evidence, participants in this study more frequently interpreted disability through spiritual beliefs, family responsibilities and culturally embedded expectations of ageing.

The use of the SPADS and CADS scales further contributes methodological value. These tools offer a structured way to quantify subjective perceptions of ageing and disability, an aspect under‐represented in prior African gerontology research. As such, the study not only confirms global patterns but also provides the first regionally grounded evidence using psychometrically validated instruments.

## 5 Conclusions

The study explored how participants in the Shai Osudoku District of the Greater Accra region, Ghana, associated symptoms of ageing with disability based on their subjective perceptions. Using the SPADS scale, it examined the influence of these perceptions on the broader conceptualisation of ageing and disability using the CADS. SPADS significantly predicted CADS, indicating that participants conceptualised ageing as being with disability. These findings confirmed the theoretical underpinning for using SPADS and CADS. Moreover, the coherence between qualitative themes and quantitative factor structures strengthens the validity of SPADS and CADS for the Dodowa population, though broader validation across Ghana is recommended.

The study also revealed nuanced roles of age, ethnicity and education, which were contrary to previous research. Living with an older adult with a disability emerged as a significant predictor and influenced perceptions, reflecting the biopsychosocial model of disability. By linking socio‐demographic factors with the conceptualisation of ageing and disability, the study enriches understanding of the relationships between ageing, disability and social context in Ghana. These perceptions underscore the need for tailored interventions and highlight the importance of considering diverse perspectives in addressing age‐related issues. Further research should explore underlying mechanisms and longitudinal changes to inform targeted interventions effectively.

Although participants in this study conceptualised ageing in ways consistent with global ageing research, this study fills an important regional gap by documenting these perceptions within a Ghanaian setting using locally validated measurement tools. The convergence between the results of this study and global evidence underscores the universality of certain ageing perceptions, whereas the contextual specificities observed highlight the value of including African voices in global ageing discourse.

## 6 Implications for Practice and Policy

### 6.1 Interventions to Alter Negative Perceptions of Ageing

The findings indicate that subjective perceptions of ageing and disability are strongly influenced by personal experience with older adults and exposure to disability. Interventions should, therefore, be targeted, culturally relevant and context‐specific:

- **Community‐Based Programmes**: Develop initiatives in local communities that facilitate positive interactions between younger adults and older adults, especially those with disabilities. These could include mentorship, shared activities and storytelling sessions highlighting active, capable older adults.
- **Culturally Sensitive Education**: Educational campaigns should incorporate local beliefs and practices, addressing misconceptions about ageing, disability and factors such as divine causation or witchcraft. Integrating these into community durbars, religious gatherings and family forums can enhance acceptability and impact.
- **Household‐Focused Engagement**: Families living with older adults with disabilities can be targeted for workshops or support groups to foster positive perceptions and coping strategies, given the association between direct exposure and higher SPADS scores.

### 6.2 Policy and Structural Recommendations

- **Use of Measurement Tools**: The SPADS and CADS scales provide a reliable framework to monitor the impact of interventions aimed at shifting perceptions of ageing and disability. Policymakers and practitioners can use these tools to track changes over time and tailor programmes accordingly.
- **Age‐Friendly Environments**: Urban planning and community infrastructure should consider accessibility for older adults, including safe walking paths, seating and lighting in public spaces, reflecting the everyday challenges noted in participants’ experiences.
- **Targeted Support Services**: Policies should prioritise home care and mental health services for older adults, particularly those living with disability, to address both physical and psychosocial needs within the Ghanaian context.

By grounding interventions and policy recommendations in the lived experiences captured by SPADS and CADS, society can promote **positive, culturally informed perceptions of ageing**, reduce stigma and improve the well‐being of older adults in Ghana.

## 7 Implications for Future Research

The study currently focuses on Dodowa, a peri‐urban area. Future research should include rural and urban samples to explore how geographical context influences perceptions of ageing and disability. Moreover, investigating differences in access to healthcare, education and social support systems will provide a more nuanced understanding of how context shapes conceptualisations and inform location‐specific interventions.

## 8 Limitations of the Study

Although the SPADS and CADS scales demonstrated strong internal consistency and model fit, further reflection is needed on their cultural fit with the study population. Although items were derived from qualitative themes in Dodowa, the extent to which they generalise to other Ghanaian or sub‐Saharan contexts remains uncertain. Additionally, the spatially systematic sampling method, though practical, may have excluded certain household types or demographic groups, potentially limiting representativeness.

Furthermore, the sample size (*n* = 175), while sufficient for psychometric validation, is modest and may limit generalisability to broader populations. Participants aged 60 and above were grouped together as ‘older adults’, which does not fully capture the heterogeneity of ageing experiences; future studies could examine narrower age subgroups. The sample was predominantly female, reflecting the study context, which provides important perspectives into women's experiences but limits representation of male perspectives. Despite these limitations, the study offers an important contribution by developing and validating context‐specific measures to guide future research on ageing and disability in sub‐Saharan Africa.

## Author Contributions

**Mumuni Abu**: conceptualisation, writing – review and editing, methodology, supervision. **Fidelia Dake**: conceptualisation, writing – review and editing, methodology, supervision. **Delali Margaret Badasu**: conceptualisation, writing – review and editing, methodology, supervision. **Doris Akosua Tay**: conceptualisation, investigation, funding acquisition, writing – original draft, methodology, validation, writing – review and editing, software, formal analysis, project administration, data curation, resources, visualisation.

## Funding

This study was supported by the Carnegie Corporation of New York through the BANGA Africa Project and by the Andrew W. Mellon Foundation through the ENCAPEH Project at the University of Ghana.

## Disclosure

The authors confirm that

- This manuscript is original and has not been published previously.
- The manuscript is not under consideration for publication elsewhere.
- All authors have approved the submitted version of the manuscript.
- All authors meet the criteria for authorship.
- The research was conducted in accordance with applicable ethical guidelines.
- The funders had no role in the study design, data collection, analysis, interpretation or writing of the manuscript.

## Ethics Statement

Ethical clearance for the study was obtained from the Ethics Committee for the Humanities, University of Ghana, with clearance number ECH 128/20‐21, valid until 8 May 2022, extended to 26 March 2024. Additionally, permission was secured from the Shai Osudoku District office. Participants aged 20 and above provided informed consent after being briefed on the study's purpose, procedures, confidentiality and their right to withdraw. COVID‐19 precautions, including mask‐wearing and social distancing, were observed during data collection. Participant data were anonymised, ensuring confidentiality, and solely used for research purposes.

## Consent

Informed consent was obtained from all participants prior to their participation in the study.

## Conflicts of Interest

The authors declare no conflicts of interest.

## Permission to Reproduce Material

No copyrighted material requiring permission has been reproduced in this manuscript.

## Supporting information




**Supporting file 1**: puh270324‐sup‐0001‐SuppMat.docx

## Data Availability

The data that support the findings of this study are available from the corresponding author upon reasonable request. Data are not publicly available because they contain information that could compromise the privacy of research participants.
