# Supplementary material for: Subjective Perceptions and Conceptualisations of Ageing and Disability in Ghana: An Exploratory Sequential Mixed‐Methods Study
Source: Public Health Chall. 2026 Aug 10;5(3):e70324. doi: 10.1002/puh2.70324 (PMC13455678; doi:10.1002/puh2.70324)
Supplement: Supplementary file 1 [file PUH2-5-e70324-s001.docx]

**Supplementary File 1**

**Supplementary Table S1:** Broader Conceptualisation of Ageing and Disability Based on SPADS, CFS, MFS, and CAD Variables

| **Initial 41 SPADS, CFS, and MFS Variables** | **16 Retained CADS Variables** |
| --- | --- |
| **SPADS**  Strange behaviour  Curses  Shouts  Rude  Talks to self  Quarrelsome  Mind turns into a child  Thinking slows  Memory loss  Gets lost  Pains  White hair  Hair loss  Return to old issues  Hearing difficulty  **CFS**  Experience from other family members  Knowledge from personal experience  Knowledge from personal growth  Fear of inability to perform life activities  Fear of mobility challenges  Fear of disease  Fear of inability to perform selfcare  Fear of excessive talking  Fear of disability  Fear of witchcraft accusation  **MFS**  Sits to bath  Cannot bath  Cannot drink or feed  Being cared for  Assisted to washroom  Cannot do laundry  Cannot cook  Cannot sweep  Cannot farm  Cannot fetch water  Being assisted  Speed in activities reduces  Cannot do previous activities  Stuck at one place  Rests between walks  Uses stick to walk | Strange behaviour  Shouts  Quarrelsome  Thinking slows  Memory loss  Pain  Sits to bath  Assisted to washroom  Cannot cook  Rests between walks  Knowledge from personal growth  Fear of inability to perform life activities  Fear of mobility challenges  Fear of disease  Fear of disability  Fear of witchcraft accusation |

**Supplementary Table S2:** Symptoms of Ageing: Participant-Identified Associations with Disability and SPADS Variables

| **26 Initial Variables** | **15 Retained SPAD Variables** |
| --- | --- |
| **Sensory impairment**  Blindness  Any eye defect  Difficulty in hearing  Deafness  Hearing and vision impairment not related to ageing and disability  **Cognition**  Gives advice  Strange `behaviour  Curses  Shouts  Rude  Talks to self  Quarrelsome  Mind turns into a child  Rate of thinking slows  Memory loss  Gets lost  Returns to old issues  **Physical attributes**  Pains  White hair  Hair loss  Amputated limb  **Likelihood of Ageing with Disability**  Disability in ageing is not possible  Everyone gets disability during ageing  Any change during ageing is a disability  If not from birth not disability  Short and smallish people age without disability | Strange behaviour  Curses  Shouts  Rude  Talks to self  Quarrelsome  Mind turns into a child  Thinking slows  Memory loss  Gets lost  Pains  White hair  Hair loss  Return to old issues  Hearing difficulty |

**Figure S1:** **Instrument Development Flowchart**

Qualitative Interviews (Dangme, Twi, English)

↓

Transcription and Validation (Native speaker cross-check for accuracy)

↓

Open Coding (In vivo and inductive coding)

↓

Axial Coding (Grouping codes into categories)

↓

Theme Integration (Cognition, Physical, Sensory, Medical, Contextual domains)

↓

Use of Qualitative Themes and Codes to Develop Questionnaire Items

↓

Pretesting and Quantitative Data Collection (n = 175; Kobo Toolbox)

↓

Item Development for SPADS, MFS, and CFS

↓

Reliability Analysis (Cronbach’s α for internal consistency)

↓

Exploratory Factor Analysis (EFA; Factor structure and item refinement)

↓

Validated Scales (SPADS, MFS, CFS)

↓

Construction of CADS (Derived from SPADS, MFS, and CFS)

↓

Structural Equation Modelling (SEM; Testing predictive relationships between SPADS and CADS)

**Mapping of Qualitative Themes to SPADS Items**

This mapping draws directly from the qualitative findings and item development process described in the study. It links thematic categories and participant insights to the final SPADS items, illustrating how subjective perceptions of ageing informed scale construction.

Theme 1: Cognition

**Categories:** Communication and Cognitive Attributes

**Codes:**

- *Communication (Negative)*: Cursing, insults, rudeness, strange behaviour, shouting, talks to self, talks like a child, excessive talking, unsound conversation
- *Communication (Positive)*: Expresses gratitude, gives advice, talks nicely, admits faults
- *Cognitive Attributes*: Memory loss, gets lost, mind turns into child, repetition of issues, returns to old issues, sobs often, excessive thinking

**Mapped SPADS Items:**

- Strange behaviour
- Curses
- Shouts
- Rude
- Talks to self
- Quarrelsome
- Mind turns into a child
- Thinking slows
- Memory loss
- Gets lost
- Return to old issues

**Illustrative Quotes:**

- "It gets to a stage that the person is ageing when his mind turns to that of a child."
- "My grandmother shouts when she is speaking to you. It is nothing bad, but it is because of her being old."
- "Someone may be sick and it affects his mind... she takes over an issue that does not concern her."

**Conceptualisation:** Ageing with disability (dominant), though some participants viewed certain behaviours as ageing without disability.

Theme 2: Physical Attributes

**Codes:**

- *Ageing with Disability*: Pains, weakness, white hair, hair loss, broken limb, deformity, head shaking, waist/knee/back problems
- *Ageing without Disability*: Strong, reduced strength, short/small body structure

**Mapped SPADS Items:**

- Pains
- White hair
- Hair loss

**Illustrative Quotes:**

- "Your knees, your side, and your entire body starts hurting."
- "Those who age without a disability are the short and smallish people."

**Conceptualisation:** Ageing with disability (especially linked to pain and physical impairment); white hair often viewed as ageing without disability.

Theme 3: Sensory Impairment

**Codes:**

- *Ageing with Disability*: Blindness, not seeing clearly, blocked ears, deafness, hurting eyes, near-sightedness, difficulty hearing
- *Ageing without Disability*: Reads without spectacles, eye defect (seen as disease)

**Mapped SPADS Item:**

- Hearing difficulty

**Illustrative Quotes:**

- "Some old people don't see well... it is a disability."
- "She reads the Bible without spectacles... someone can grow without a disability."

**Conceptualisation:** Predominantly ageing with disability, though some participants distinguished impairment from disability.

Theme 4: Possibility of Disability During Ageing

**Codes:**

- *Views*: Everyone gets disability, disability occurs at any age, ageing occurs with or without disability, disability is unpredictable

**Mapped SPADS Items:**

- Return to old issues
- Curses

**Illustrative Quotes:**

- "Disability is part of it... maybe it's God."
- "If it is not on you, it is not on you... I will age without it."

**Conceptualisation:** Mixed- some participants viewed disability as inevitable with ageing (medical model), others saw it as context-dependent (biopsychosocial model).

This mapping demonstrates the direct link between qualitative insights and SPADS item development, reinforcing the cultural and experiential relevance of the scale.

**Mapping of Qualitative Themes to CADS Items**

This mapping integrates qualitative insights from SPADS, MFS, and CFS to show how the Conceptualisation of Ageing and Disability Scale (CADS) items were derived. It includes thematic categories, participant quotes, and conceptualisation direction.

SPADS-Derived Items (Cognition)

**Themes:** Communication and Cognitive Attributes

**Mapped CADS Items:**

- Strange behaviour
- Shouts
- Quarrelsome
- Thinking slows
- Memory loss
- Pain

**Illustrative Quotes:**

- "It gets to a stage that the person is ageing when his mind turns to that of a child."
- "My grandmother shouts when she is speaking to you. It is nothing bad, but it is because of her being old."

**Conceptualisation:** Ageing with disability (dominant view)

MFS-Derived Items (Medical Factors)

**Themes:** Diseases, Health Decline, Performance of ADLs and IADLs

**Mapped CADS Items:**

- Sits to bath
- Assisted to washroom
- Cannot cook
- Rests between walks

**Illustrative Quotes:**

- "Because of the disease she cannot do that. She used to farm, now she can't."
- "You have to rest before you continue walking."
- "You cannot bath, cannot go to the washroom... someone must do it for you."

**Conceptualisation:** Strongly ageing with disability (medical model dominant)

CFS-Derived Items (Contextual Factors)

**Themes:** Fear, Social Participation, Beliefs, Experience

**Mapped CADS Items:**

- Knowledge from personal growth
- Fear of inability to perform life activities
- Fear of mobility challenges
- Fear of disease
- Fear of disability
- Fear of witchcraft accusation

**Illustrative Quotes:**

- "I plead God... that I cannot go to the washroom to pass urine or do something."
- "You start having menopause, talking... then they start calling you witch."
- "If you live a good life nothing will happen to you."

**Conceptualisation:** Mixed some fears and beliefs linked to ageing with disability, others reflect social or biopsychosocial models.

This mapping shows how CADS items were grounded in lived experiences and culturally relevant perceptions of ageing and disability, ensuring conceptual and contextual validity.

**Table S3: SPADS Items for Replication**

| **Item Number** | **Item Description** | **Thematic Domain** | **Response Format** | **Scoring Instructions** | **Interpretation** |
| --- | --- | --- | --- | --- | --- |
| 1 | Strange behaviour | Cognition | 3-point Likert | 3 = Agree, 2 = Neutral, 1 = Disagree | Higher score = stronger belief ageing = disability |
| 2 | Curses | Cognition | 3-point Likert | 3 = Agree, 2 = Neutral, 1 = Disagree | Lower score = ageing seen as distinct from disability |
| 3 | Shouts | Cognition | 3-point Likert | 3 = Agree, 2 = Neutral, 1 = Disagree | Higher score = ageing linked to disability |
| 4 | Rude | Cognition | 3-point Likert | 3 = Agree, 2 = Neutral, 1 = Disagree | Higher score = ageing linked to disability |
| 5 | Talks to self | Cognition | 3-point Likert | 3 = Agree, 2 = Neutral, 1 = Disagree | Higher score = ageing linked to disability |
| 6 | Quarrelsome | Cognition | 3-point Likert | 3 = Agree, 2 = Neutral, 1 = Disagree | Higher score = ageing linked to disability |
| 7 | Mind turns into a child | Cognition | 3-point Likert | 3 = Agree, 2 = Neutral, 1 = Disagree | Higher score = ageing linked to disability |
| 8 | Thinking slows | Cognition | 3-point Likert | 3 = Agree, 2 = Neutral, 1 = Disagree | Higher score = ageing linked to disability |
| 9 | Memory loss | Cognition | 3-point Likert | 3 = Agree, 2 = Neutral, 1 = Disagree | Higher score = ageing linked to disability |
| 10 | Gets lost | Cognition | 3-point Likert | 3 = Agree, 2 = Neutral, 1 = Disagree | Higher score = ageing linked to disability |
| 11 | Pains | Physical attributes | 3-point Likert | 3 = Agree, 2 = Neutral, 1 = Disagree | Higher score = ageing linked to disability |
| 12 | White hair | Physical attributes | 3-point Likert | 3 = Agree, 2 = Neutral, 1 = Disagree | Lower score = ageing seen as distinct from disability |
| 13 | Hair loss | Physical attributes | 3-point Likert | 3 = Agree, 2 = Neutral, 1 = Disagree | Lower score = ageing seen as distinct from disability |
| 14 | Return to old issues | Cognition | 3-point Likert | 3 = Agree, 2 = Neutral, 1 = Disagree | Higher score = ageing linked to disability |
| 15 | Hearing difficulty | Sensory impairment | Binary | 3 = Selected 1 = Not selected | Higher score = ageing linked to disability |

**Table S4: CADS Items for Replication**

| Item Number | Item Description | Thematic Domain | Response Format | Scoring Instructions | Interpretation |
| --- | --- | --- | --- | --- | --- |
| 1 | Strange behaviour | Cognition | 3-point Likert | 3 = Agree, 2 = Neutral, 1 = Disagree | Higher score = ageing conceptualised with disability |
| 2 | Shouts | Cognition | 3-point Likert | 3 = Agree, 2 = Neutral, 1 = Disagree | Higher score = ageing conceptualised with disability |
| 3 | Quarrelsome | Cognition | 3-point Likert | 3 = Agree, 2 = Neutral, 1 = Disagree | Higher score = ageing conceptualised with disability |
| 4 | Thinking slows | Cognition | 3-point Likert | 3 = Agree, 2 = Neutral, 1 = Disagree | Higher score = ageing conceptualised with disability |
| 5 | Memory loss | Cognition | 3-point Likert | 3 = Agree, 2 = Neutral, 1 = Disagree | Higher score = ageing conceptualised with disability |
| 6 | Pain | Medical factors | 3-point Likert | 3 = Agree, 2 = Neutral, 1 = Disagree | Higher score = ageing conceptualised with disability |
| 7 | Sits to bath | Medical factors | Binary | 3 = Selected, 1 = Not selected | Higher score = ageing conceptualised with disability |
| 8 | Assisted to washroom | Medical factors | Binary | 3 = Selected, 1 = Not selected | Higher score = ageing conceptualised with disability |
| 9 | Cannot cook | Medical factors | Binary | 3 = Selected, 1 = Not selected | Higher score = ageing conceptualised with disability |
| 10 | Rests between walks | Medical factors | 3-point Likert | 3 = Agree, 2 = Neutral, 1 = Disagree | Higher score = ageing conceptualised with disability |
| 11 | Knowledge from personal growth | Contextual factors | Binary | 3 = Selected, 1 = Not selected | Higher score = ageing conceptualised with disability |
| 12 | Fear of inability to perform life activities | Contextual factors | Binary | 3 = Selected, 1 = Not selected | Higher score = ageing conceptualised with disability |
| 13 | Fear of mobility challenges | Contextual factors | Binary | 3 = Selected, 1 = Not selected | Higher score = ageing conceptualised with disability |
| 14 | Fear of disease | Contextual factors | Binary | 3 = Selected, 1 = Not selected | Higher score = ageing conceptualised with disability |
| 15 | Fear of disability | Contextual factors | Binary | 3 = Selected, 1 = Not selected | Higher score = ageing conceptualised with disability |
| 16 | Fear of witchcraft accusation | Contextual factors | Binary | 3 = Selected, 1 = Not selected | Higher score = ageing conceptualised with disability |
